# Supplementary material for: Structural Analysis and Optimization of Convolutional Neural Networks with a Small Sample Size
Source: Sci Rep. 2020 Jan 21;10:834. doi: 10.1038/s41598-020-57866-2 (PMC6972775; doi:10.1038/s41598-020-57866-2)
Supplement: Supplementary file 1 — Supplementary Information. [file 41598_2020_57866_MOESM1_ESM.docx]

**Structural Analysis and Optimization of Convolutional Neural Networks with a Small Sample Size**

Rhett N. D’souza^1^, Po-Yao Huang^2^, Fang-Cheng Yeh^3,^*

^1^Department of Electrical Engineering and Computer Science, McCormick School of Engineering and Applied Science, Northwestern University, Evanston IL, USA

^2^Language Technologies Institute, School of Computer Science, Carnegie Mellon University, Pittsburgh PA, USA

^3^Department of Bioengineering, University of Pittsburgh, Pittsburgh PA, USA

# Supplementary Information

The classification accuracy is the percent fraction of examples the network correctly predicts for the classification task. The classification error % indicates the rate of miss-classifications for a specific task. This classification error is equal to 100% subtracted by the classification accuracy.

In this context, the term “channel” refers to a specific single layer in the network architecture. The width is the number of nodes in a specific layer/channel. The depth is the total number of layers present in the network. A filter indicates a specific small unit that is used during the convolution and pooling operations on a layer’s input images/representations. The kernel size indicates the dimensions of this filter. For example, a kernel size of 5 indicates a 5x5 size filter that is used for the convolution and/or pooling operation.

The validation error, in this case, was calculated using the validation set, which referred to a per epoch holdout set for monitoring of training. After each epoch of training, the network was validated with this holdout set and the performance was registered. This validation holdout set was different from the final test set, which included a set of samples that were never looked at by the network. This validation set was used for the task of understanding at which state the network was best trained, as well as implementing early stopping to avoid overfitting.
